# Computational Models Reveal a Passive Mechanism for Cell Migration in the Crypt 

**DOI:** 10.1371/journal.pone.0080516

**Published:** 2013-11-18

**Authors:** Sara-Jane Dunn, Inke S. Näthke, James M. Osborne

**Affiliations:** 1 Computational Science Laboratory, Microsoft Research Ltd., Cambridge, United Kingdom; 2 Division of Cell and Developmental Biology, University of Dundee, Dundee, United Kingdom; 3 Department of Computer Science, University of Oxford, Oxford, United Kingdom; University of Erlangen-Nuremberg, Germany

## Abstract

Cell migration in the intestinal crypt is essential for the regular renewal of the epithelium, and the continued upward movement of cells is a key characteristic of healthy crypt dynamics. However, the driving force behind this migration is unknown. Possibilities include mitotic pressure, active movement driven by motility cues, or negative pressure arising from cell loss at the crypt collar. It is possible that a combination of factors together coordinate migration. Here, three different computational models are used to provide insight into the mechanisms that underpin cell movement in the crypt, by examining the consequence of eliminating cell division on cell movement. Computational simulations agree with existing experimental results, confirming that migration can continue in the absence of mitosis. Importantly, however, simulations allow us to infer mechanisms that are sufficient to generate cell movement, which is not possible through experimental observation alone. The results produced by the three models agree and suggest that cell loss due to apoptosis and extrusion at the crypt collar relieves cell compression below, allowing cells to expand and move upwards. This finding suggests that future experiments should focus on the role of apoptosis and cell extrusion in controlling cell migration in the crypt.

## Introduction

**Figure 1:**
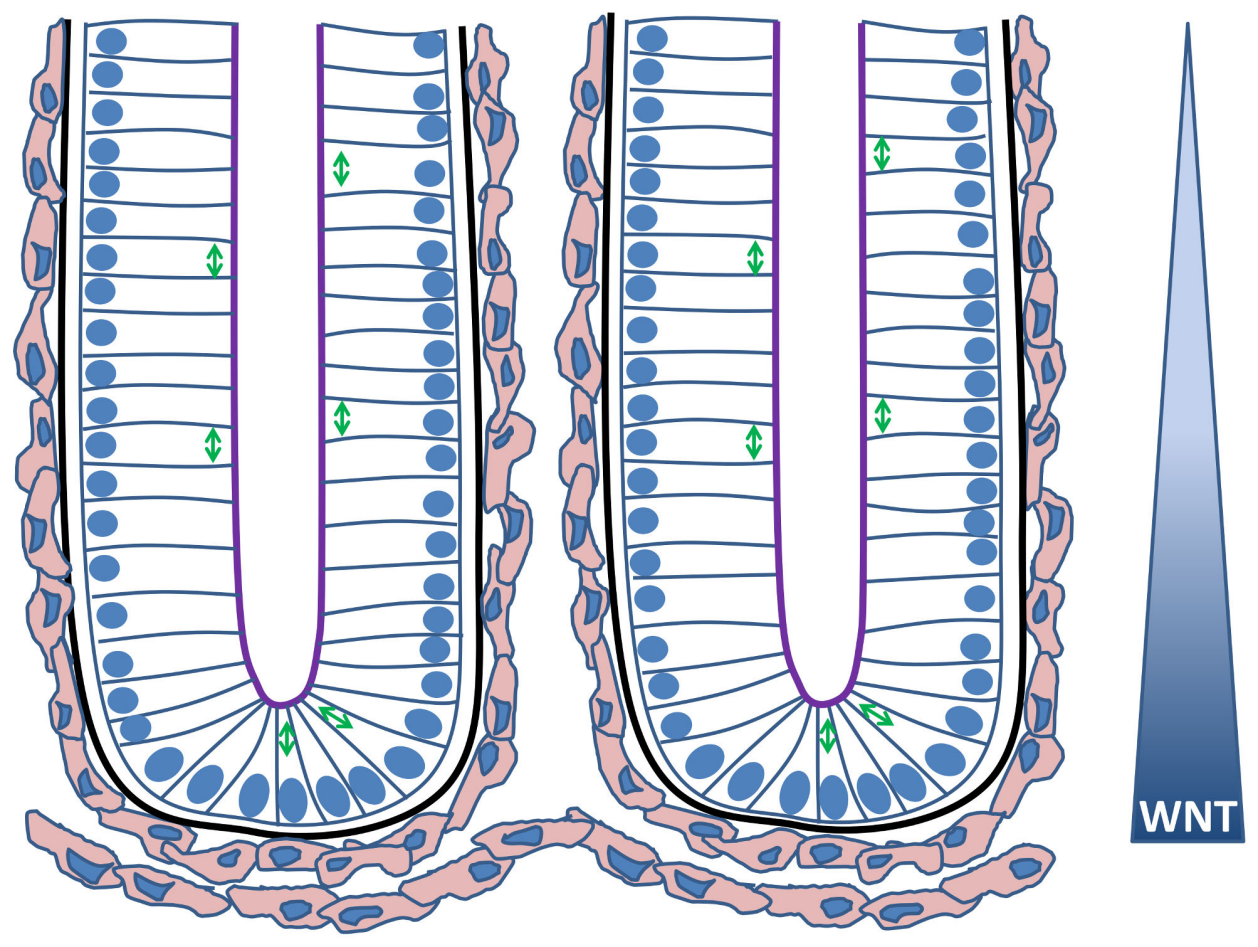
A cartoon sketch illustrating two neighbouring crypts. The nuclei of the epithelial cells are indicated in blue, and the arrows illustrate the typical alignment of the mitotic spindle during division for various cell positions. The apical surface of each epithelial cell faces the crypt lumen (purple) while the basal surface is in contact with the basement membrane (black). The myofibroblasts that form the pericryptal fibroblast sheath are coloured pink. A decreasing gradient of Wnt signalling factors exists along the crypt axis, influencing the proliferative state of the epithelial cells.

The intestinal epithelium is the most rapidly regenerating surface in the human body, with a renewal process that is coordinated by glands known as the crypts of Lieberkühn. This process requires synchronised cell proliferation, migration, differentiation and cell loss. Crypts are closely packed, test-tube shaped invaginations that regularly punctuate the surface of the intestine (Figure 1). Each crypt is lined with a monolayer of contiguous epithelial cells anchored to a basement membrane. These epithelial cells exist in a proliferative hierarchy of stem, transit-amplifying and differentiated cells that include absorptive and secretory cells [1]. Within the small intestine, a cluster of crypts feeds directly onto single villi, which project outwards into the lumen of the gut. In contrast, the surface of the large intestine is largely flat, consisting only of crypts. 

Crypt homeostasis is regulated by key signalling pathways. Wnt signalling drives cell proliferation, and a decreasing gradient of Wnt along the crypt axis correlates with decreasing “stemness” [2]. Notch signalling is also essential to maintain the proliferative compartment in the crypt, and has a dual role in specifying cell fates towards either an absorptive or secretory cell type [3]. The combination of Wnt and Notch signals is key for maintaining proliferation. BMP signalling increases along the crypt axis and is likely involved in crypt branching and differentiation [4]. Finally, cell sorting/positioning is regulated by Eph/ephrin signalling between neighbouring cells, and each position along the crypt-villus axis is characterised by different levels of EphB and ephrin-B molecules [5,6].

Directed migration of cells occurs from the proliferative compartment towards the crypt collar. Paneth cells in the small-intestinal crypts are the exception and migrate towards or remain near the crypt base, where they reside interspersed between stem cells. Once cells reach the crypt collar, or the villus tip, they are shed into the gut lumen so that the overall lifetime of a cell is approximately 60 hours [7,8]. The upward migration and regular clearance of cells from crypts provides the gut with a level of protection because it ensures that cells that acquire oncogenic mutations cannot remain long enough to disrupt homeostasis. One implication of this situation is that for a mutation to ensure its propagation in the crypt, it must decrease normal migration. This could be achieved by changing the direction and/or rate of cell migration, or by changing responses to apoptosis cues [9], and the implications of such changes have been investigated computationally [10,11]. Understanding cell migration in the crypt is key for further research into the onset and development of colorectal cancer (CRC), and to developing effective therapies. 

Inactivation of the Adenomatous Polyposis Coli (Apc) tumour suppressor gene is the initiating event in most sporadic CRCs [12,13]. In addition, heterozygous germline mutation in *Apc* is responsible for the heritable condition Familial Adenomatous Polyposis (FAP). FAP patients develop numerous benign growths in normal-appearing colonic mucosa, which progress into CRC, usually much earlier than in sporadic cases [14,15]. The mechanisms responsible for the cancerous changes induced by *Apc* mutations involve its role as a scaffold protein in a destruction complex for β-catenin. Loss of APC activates the canonical Wnt pathway by stabilising β-catenin, which causes translocation of β-catenin to the nucleus, where it acts as a transcriptional activator [16]. This leads to increased proliferation, a failure to differentiate and also impacts on cell migration [17]. In addition, the stabilising effect of APC on cytoskeletal proteins means that loss of APC also directly causes defects in cell migration [18]. 

To examine the effect of *Apc* mutation on cell migration, Nelson et al. [19] recorded cells in live gut tissue from wildtype and *Apc* ^Min/+^ heterozygote mice. Their results revealed a dominant effect of truncated *Apc* in the *Apc* ^Min/+^ precancerous epithelial cells, which exhibit a lack of directionality in their migration. It is possible that this is caused by changes in cytoskeletal processes regulated by APC. Such investigations that measure cell migration are key for the development of CRC therapies - for example, decreased cell migration in *Apc* ^Min/+^ mice can be restored by treatment with sulindac sulfide, and this correlates with intestinal tumour regression in humans [20].

The crypt is a prime example of a tissue whose behaviour can be described using mathematical and computational approaches to generate important insights. As described above, many elements of its biology are well characterised, and it is a relatively small, compact system with well-defined functions. A number of mathematical and computational models have provided insight into different aspects of crypt dynamics. More recently, these models have adopted multiscale frameworks to inform understanding of processes occurring across multiple spatial scales. The earliest of these models utilised cellular automata on a rigid, cylindrical approximation of the crypt geometry to define individual cells as equally-sized lattice sites [21–23]. Cell positions evolved according to specific spatial rules. Results from this type of modelling work have, for example, explained the observed distribution of goblet cells in the crypt [24]. The closely related cellular Potts model [25], which defines single cells as multiple, adjacent lattice sites and evolves cell movement subject to energy optimisation constraints, has been used to examine differential adhesion due to Eph/ephrin signalling between neighbouring cells and suggests that differential adhesion is both a key regulator of cell sorting, as well as coordinated, directed cell migration [26].

To address the restrictions imposed by a fixed lattice of cell locations, more recent models have adopted lattice-free frameworks, such as the cell-centre tessellation model [27–30], the cell-vertex model [10] and the overlapping spheres model [31–33]. In each case, cell movement is continuous and free in space, and cells move due to forces exerted by neighbouring cells. Such models have examined lineage specification and crypt monoclonality. The role of crypt geometry has also been considered, both in a two-dimensional (2D) cross-sectional deformable geometry [34], and a 3D test-tube shaped, rigid geometry [35], and more recently in a computational model of the self-organising crypt-villus organoids [36]. It is suggested that the geometry of the crypt leads to the compression of cells at the curved crypt base, which can force a perpendicular alignment of the mitotic spindle during division events [34]. Furthermore, the same modelling work suggests that cell extrusion towards the top of the crypt may be a consequence of cell overcrowding, as well as the negative curvature at the collar that reduces the contact area with the basement membrane. Live cell extrusion is common at the crypt collar and intercrypt table: it is a key feature of normal tissue homeostasis and is required to maintain cell numbers [8].

The aim of this report is to use computational modelling to generate new insight into the mechanisms that control cell migration in the crypt. While the causes of migration are currently unknown, mechanisms that have been proposed include: passive mechanisms such as mitotic pressure, pushing cells upwards in columns [27]; negative pressure due to cell loss at the villus tip or crypt collar [34,37]; global movement of the components of the extracellular matrix akin to ‘conveyor belt’ action of the basement membrane [38]; and, active movement due to gradients of signalling factors or adhesion systems, such as epidermal growth factor (EGF) and Ephs and ephrins [5,39].

There is evidence to suggest that pressure created by cell division in the lower half of the crypt alone cannot account for cell migration. Firstly, junctional complexes between epithelial cells should prevent extensive movement between lateral neighbours, and therefore mitotic pressure is unlikely to induce columnar movement [37]. Secondly, while circadian rhythms in migration velocity both within crypts and on small intestinal villi have been measured, there is no direct correlation between migration rate and the number of mitotic events that occur within a 24 hour period [40]. Thirdly, eliminating cell division in murine crypts using radiation and toxic agents does not abolish migration despite the lack of mitotic activity [41].

Movement of the extracellular matrix as a contributing factor to epithelial cells migration is unlikely based on the asynchronous behaviour of pericryptal fibroblasts sheet (PCFS) and epithelium [42], and because the basement membrane does not renew itself sufficiently rapidly, nor is it strong enough to carry epithelial cells upwards. Evidence indicates, instead, that the PCFS is a stable structure that epithelial cells use as a substrate for their migration [37].

Currently there is little experimental evidence for the negative pressure theory, which describes an upwards movement induced by cell loss/apoptosis at the crypt collar. However, computational modelling suggests that cell migration may be mediated by the balance between cell loss and cell division [34]. Specifically, physical pressure exerted by tight cell packing could inhibit division and cell loss near the collar could generate additional space for cells below to expand and migrate upwards. This release could relay downwards to allow mitosis. In this scenario, cell positioning would be supported by known signalling pathways, e.g. Eph/ephrin signalling.

To increase our understanding of migration in the crypt, we use three different computational models to investigate the consequence of eliminating cell division within the crypt. These results are compared with those obtained in biological experiments conducted by Kaur and Potten [41]. The components of each computational model are precisely defined, such that the mechanism for cell movement is easily inferred, rather than only observed. Each model is kept deliberately simple to investigate the contributions of only mechanical processes to migration – no active migration is assumed, and cells are either proliferative or differentiated. Three models are used to demonstrate that the results are robust, and not model-dependent. 

## Results

### Model Comparison

**Figure 2:**
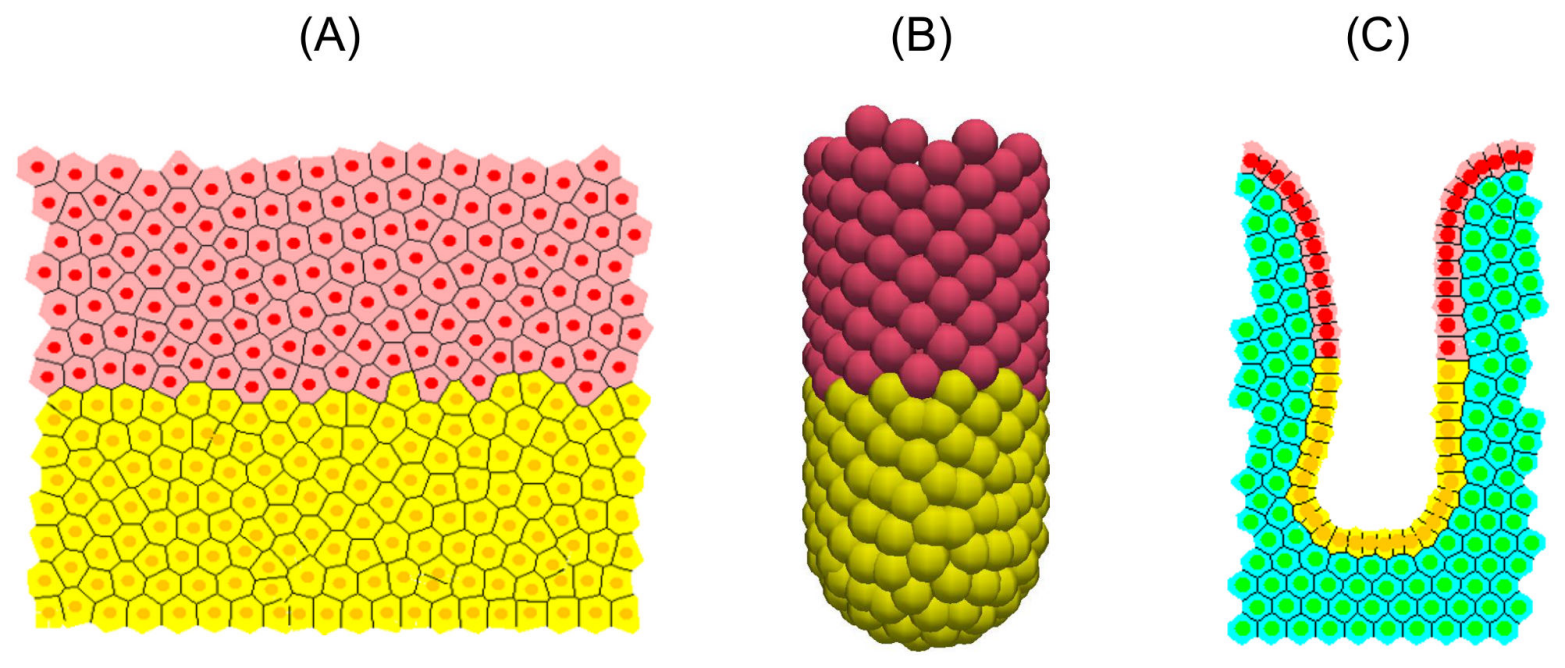
Simulation snapshots for each crypt model at steady state. Proliferative cells are coloured yellow, differentiated cells are coloured pink, and in (C), stromal cells are coloured green.

This section presents a comparison of the stable, steady state behaviour of three computational models, each described in the Materials and Methods section: a 2D cylindrical model [10]; a 3D rigid, test-tube shaped model (inspired by [35]); and a 2D deformable cross-sectional model [34]. Simulation snapshots of each model are shown in Figure 2, model parameters are given in Table 1, and the key differences between the models are summarised in Table 2. 

**Table 1:** The parameters used in each model.

	**Description**	**Cylindrical Model**	**3D Model**	**Cross-Sectional Model**
**µ**	Spring constant	30 N Cell diameter^-1^	30 N Cell diameter^-1^	45 N Cell diameter^-1^
**η**	Drag coefficient	1 N hours Cell diameter^-1^	1 N hours Cell diameter^-1^	1 N hours Cell diameter^-1^
**s**	Equilibrium spring rest length	1 Cell diameter	1 Cell diameter	1 Cell diameter
**β**	Basement membrane force parameter	--	--	12 N Cell diameter^-1^
***κ_s_***	Spontaneous curvature for crypt base	--	--	0.3 Cell diameter^-1^
***A_T_***	Threshold area for cell division	--	--	0.5 Cell diameter^2^
***A*_0_**	Equilibrium area of a cell	$\frac{\sqrt{3}}{2}$Cell diameter^2^	$\frac{\sqrt{3}}{2}$Cell diameter^2^	$\frac{\sqrt{3}}{2}$Cell diameter^2^
***ΔT***	Timestep	0.005 hours	0.005 hours	0.0042 hours
***H_T_***	Threshold height for cell sloughing	12 Cell diameter	12 Cell diameter	0.9 of total crypt length (variable)
***W_T_***	Wnt threshold that defines the proliferative compartment	0.65	0.65	0.65

**Table 2:** A brief summary of the main differences between the models and their behaviour, and how this compares to experimentally-observed behaviour of the crypt.

	**2D Cylindrical Model**	**3D Model**	**2D Cross-Sectional Model**	**Experimental System**
**Cell dynamics**	Off-lattice cell centre model			--
**Cell-cell connectivity**	Delaunay triangulation	Overlapping spheres	Delaunay triangulation	--
**Geometry**	A cylinder that is cut open and rolled out to form a 2D rectangular domain.	A rigid 3D test-tube shaped geometry.	A deformable 2D longitudinal cross-section through the crypt.	--
**Cell cycle**	Stochastic Wnt-dependent cell cycle model			--
**Cell death**	Cell sloughing is defined to occur above a threshold height.	Cell sloughing is defined to occur above a threshold height.	(i) Random cell sloughing at the crypt orifice and (ii) cell death after loss of contact with basement membrane.	Cells are sloughed towards the crypt orifice, and undergo apopotosis following extrusion from the epithelium.
**Cell velocity**	Increasing up the vertical axis, levelling out in differentiated compartment.	Increasing up the vertical axis towards the orifice.	Increasing up the vertical axis, before levelling out then decreasing on the intercrypt table.	Increases linearly moving up the crypt axis. Rate of increase slows on the villus.
**Following elimination of cell division**	Cell migration continues. Cell number decreases exponentially before plateauing. Crypt size unaffected (due to geometry).	Cell migration continues. Cell number decreases exponentially before plateauing. Crypt size unaffected (due to geometry).	Cell migration continues. Cell number decreases linearly. The crypt shrinks.	Cell migration continues, crypts shrink.

The results demonstrate that under ‘normal’ conditions, the models produce the behaviour that is expected of the crypt, and are in agreement with existing experimental data. Simulations were run for 1000 cell hours from the point of reaching a homeostatic steady state (hereafter referred to simply as steady state), where proliferation, migration and cell death are balanced, to generate a realistic representation of model stability and evolution over long time periods. For reference, a cell hour refers to the timescale of the cell cycle, not the computational runtime, and the y-axis is taken to be the longitudinal axis of the crypt in each model.

### Cell Number

**Figure 3:**
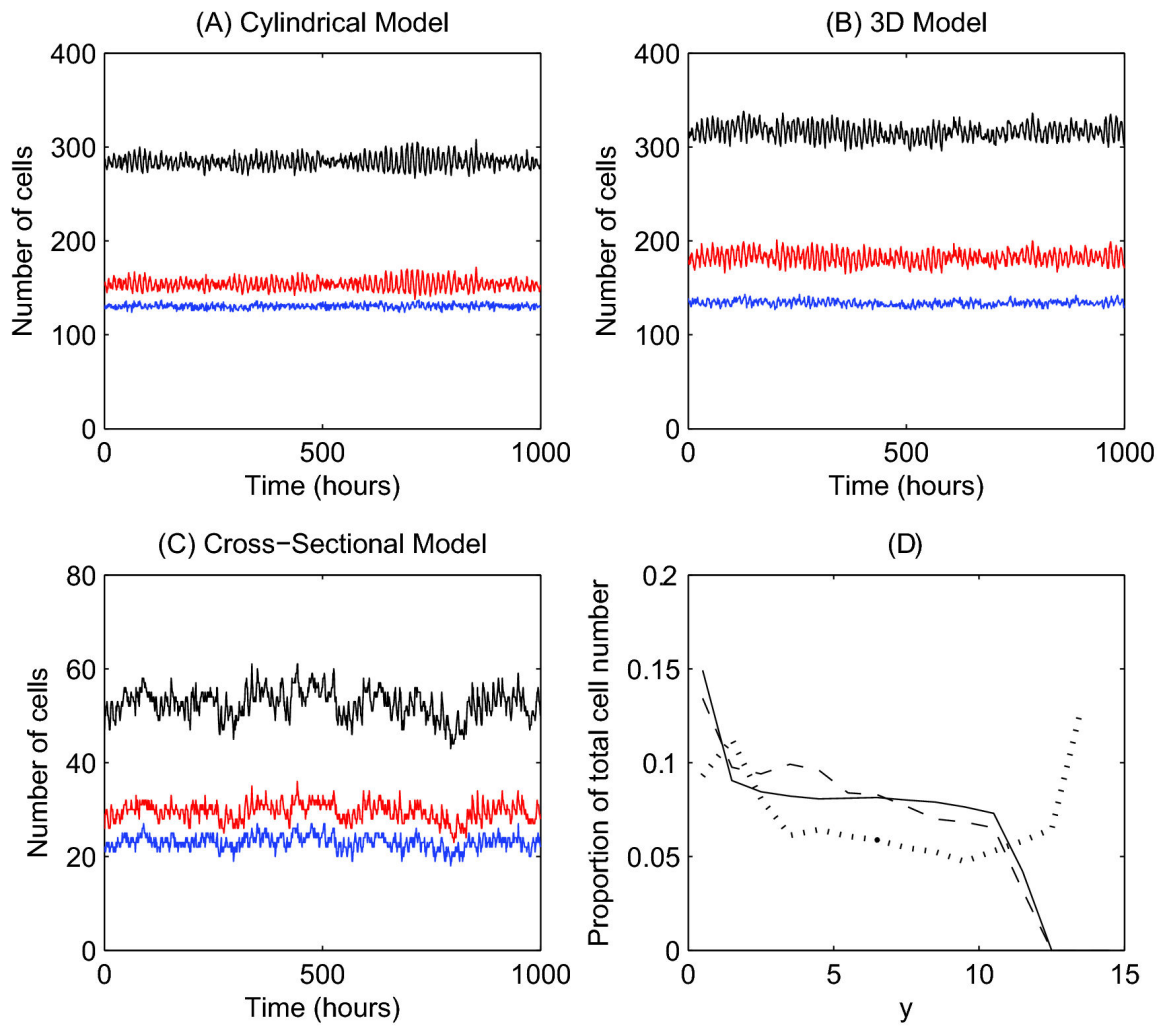
Epithelial cell number at steady state for each of the computational models. (A) - (C) Comparison of epithelial cell number at steady state for each model: the total number of epithelial cells (black) and subdivided into the number of proliferative (red) and non-proliferative cells (blue). (D) The average number of cells within bands of width 1 cell diameter for the 2D cylindrical model (solid line) and 3D rigid model (dashed line), and cross-sectional model (dotted line).

Figures 3(A) - (C) plot the total number of epithelial cells in each model over time, and show how this total is distributed into proliferative and non-proliferative cells. Each model generates behaviour that is expected for the crypt: a steady turnover of cells, such that cell birth and death are matched, and approximately half of the cells are proliferative. Stochasticity arises in all of the models primarily through random cell birth events, which are determined by a stochastic cell cycle model. In the cross-sectional model, additional stochasticity is introduced through random apoptosis events at the crypt collar, to model sloughing. The relative fluctuation in cell number for the cross-sectional model is larger than for the cylindrical and 3D models. This is due to the smaller number of cells, which generates a larger relative multiplicative noise.

Figure 3(D) plots the proportion of total epithelial cell number within horizontal bands of width 1 cell diameter: 0<*y*≤1;1<*y*≤2;…;14<*y*≤15, where *y* = 0 marks the lowest point of the crypt base. (As stated in Materials and Methods, the crypts are approximately 15 cells in height for the murine small intestine.) In each model, a high proportion of cells are observed at the crypt base, which levels out in the central portion of the crypt. It must be noted that the cylindrical model does not account for the tapered, bowl-shaped crypt base, and the larger number of cells at the base is due to cell compression there. In addition, the cross-sectional model demonstrates a second peak at *y*≈13, which coincides with the crypt collar and intercrypt table, where the monolayer is more horizontal (Figure 2(C)). Thus, while each model generates a steady cell turnover, differences in cell distribution arise due to the geometry that is used.

### Cell Area / Volume

**Figure 4:**
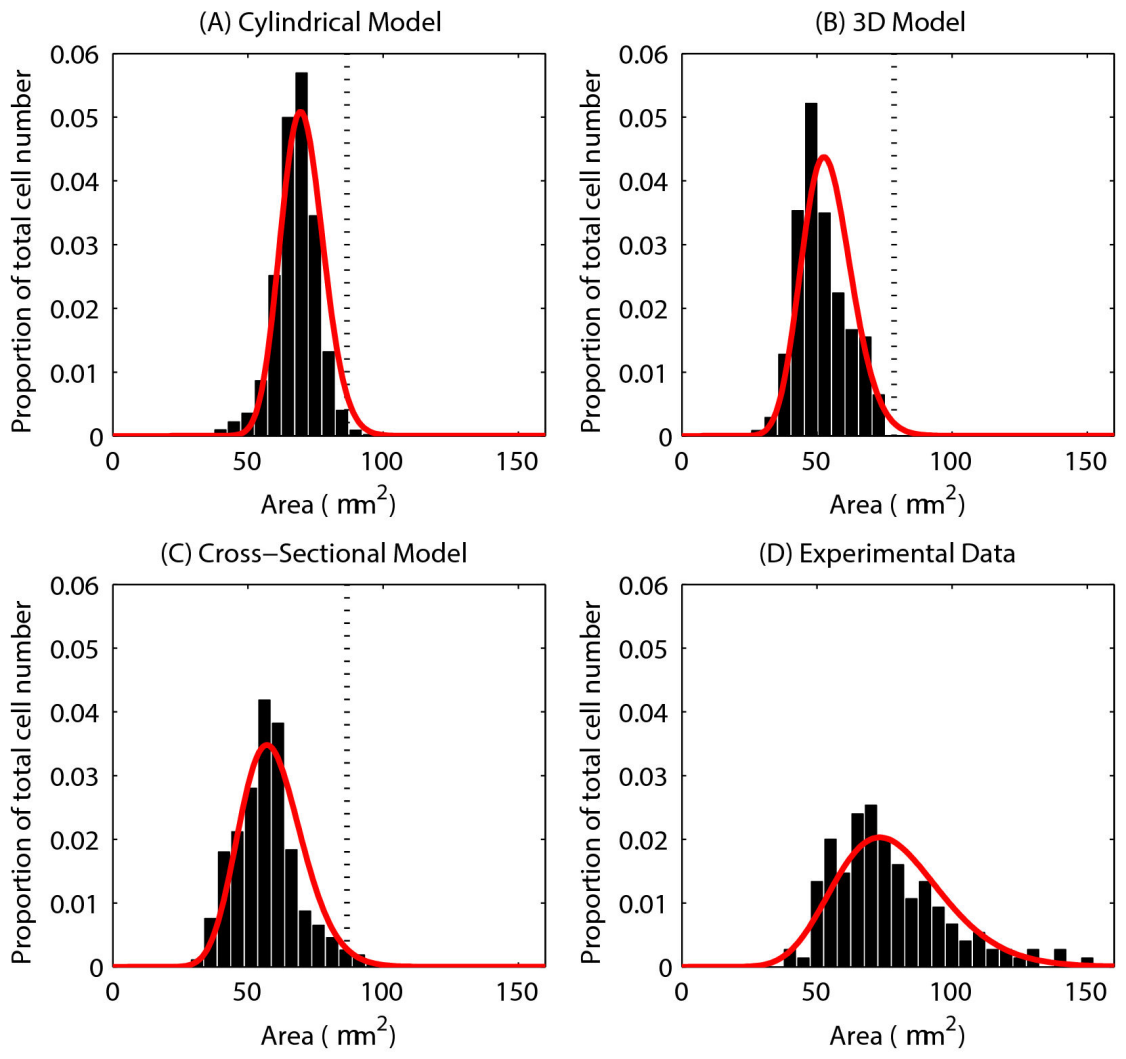
Epithelial cell size for each of the computational models. (A) - (C) The epithelial cell area distribution for each model – the red curve marks the fitted Gamma distribution in each case. The vertical dotted line marks the equilibrium area (86.6 μm^2^), and the proportion of the total number of epithelial cells is indicated on the y-axis. (D) Experimental data obtained from three wildtype murine tissue samples, taken from midway down the length of the colon. ((C) and (D) re-plotted from data provided in [34]).

Figures 4(A) - (C) show the distribution of cell areas for each of the models, where the cross-sectional cell area has been inferred from cell volume for the 3D model, assuming a circular cross-section. The red curve on each plot is a Gamma distribution fit of the data. While the models differ in their dimensions, these plots demonstrate the variation in cell size. The axes for each of the plots have been chosen to allow comparison with experimental data, shown in Figure 4(D).

The equilibrium cell size is marked on each plot by a dotted line, showing that the majority of cells are under compression in each model. Figure 4(D), replotted from [34], shows the distribution of cell areas in three wildtype murine tissue samples. Comparing the simulation results with the experimental data shows that the distributions for the 2D models ((A) and (C)) match well, with similar peaks between 50 - 75 μm^2^. In addition, the equivalent cross-sectional area of the spheres in the 3D model peaks at approximately 50 μm^2^. Thus, the turnover and cell numbers in all three models lead towards a plausible and realistic distribution of cell sizes. 

### Cell Velocity

**Figure 5:**
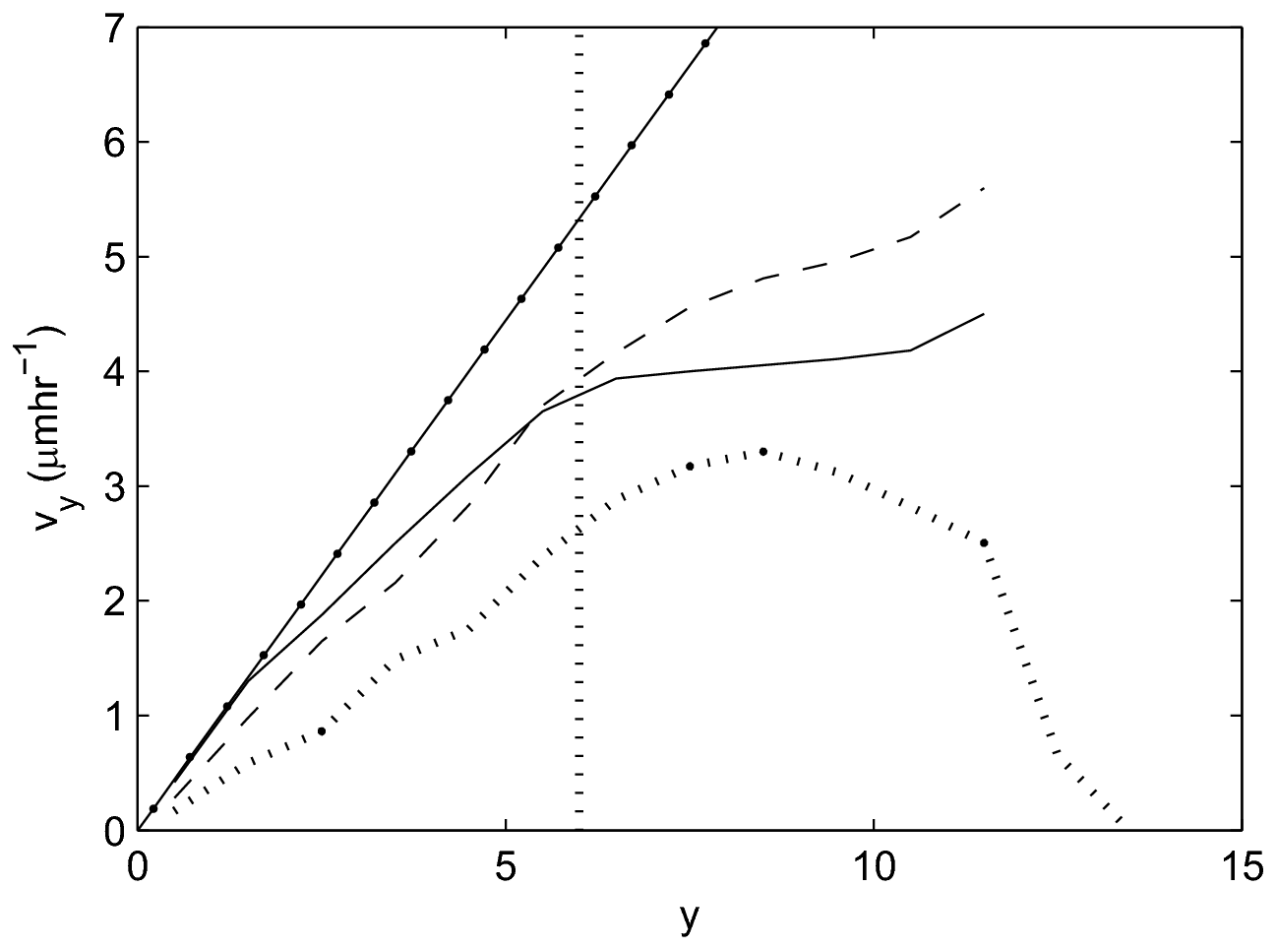
Average epithelial cell velocity, $v_{y}$, along the longitudinal crypt axis for each model. 2D cylindrical model (solid line), 3D rigid model (dashed line), cross-sectional model (dotted line), experimental data from [41] (dot-dashed line). The vertical dotted line marks the highest point of the proliferative compartment.

Figure 5 compares the upward migration velocity, *v*_*y*_, of the epithelial cells for each model with experimental data re-plotted from [41]. To generate this plot from the simulation results, *v*_*y*_ is again averaged within horizontal bands of width 1 cell diameter, where *y*=0 corresponds to the lowest point at the crypt base. The vertical line in this plot marks the highest point of the proliferative compartment. 

These results are in agreement with the linear increase in migration velocity of cells moving up the crypt-villus axis obtained experimentally. In the small intestinal crypts, a linear increase in cell velocity is observed moving towards the crypt collar, but the rate of increase in velocity decreases on the villus. In each model, the upward velocity increases along the vertical crypt axis towards the differentiated cell compartment, in agreement with experimental results. However, different behaviour is observed between the models in the differentiated compartment zone. In the cylindrical model, the velocity levels out, but in the 3D model the velocity continues to increase, and in the cross-sectional model, the velocity first levels out and then decreases. The difference in behaviour between the models is due to the difference in geometry, and it should be noted that none of the models currently take account of the villus.

In the cylindrical model, proliferative pressure is dispersed moving upwards because the cells are free to move on a plane. The differentiated cells therefore experience a smaller upward force than the proliferative cells, and hence move more slowly. In contrast, cells in the 3D model are much more compressed to begin with (Figure 4(B)), such that cell loss translates into a larger upward movement. In addition, in the 3D model there are fewer restrictions placed on cell neighbours, and therefore cell movement, when compared with the 2D models in which cell neighbours are defined by a triangulation (see Materials and Methods). In the cross-sectional model, cell velocity decreases at the crypt collar and intercrypt table, where migration is predominantly horizontal and cell loss is frequent. Including the villus in the cross-sectional model would eliminate this decrease at the crypt collar, but move it to the villus tip. These results emphasise that using a more realistic crypt geometry in a model can produce cell movement that more closely matches that observed in real tissue. This, in turn, is crucial for the value and accuracy of model predictions about processes that govern migration. 

### Kaur and Potten Migration Experiments

Kaur and Potten [41] demonstrate experimentally that movement in the crypt is not solely due to mitotic pressure arising from cell division in the lower proliferative compartment. However, the mechanism or mechanisms that underpin cell migration are not yet known. It is possible that movement could be stimulated by the presence of signalling gradients along the vertical axis, or by increased activity of ‘motility molecules’ [39]. To address this question, we briefly describe the results of the biological experiments, and those of equivalent *in silico* experiments using each of the three computational models.

In their study, Kaur and Potten applied radiation and cytotoxic agents to murine crypts, excised from mice aged between 10-13 weeks. Groups of four replicates were injected with tritiated thymidine (^3^HTdR) to label dividing cells, and radiation was applied shortly thereafter to reduce the mitotic index to zero rapidly, halting proliferation completely for at least 8 hours (cell death still occurred). Mitotic activity did not return to the pre-irradiation level over the first 12 hours of treatment, but began to increase after 8 hours.

**Figure 6:**
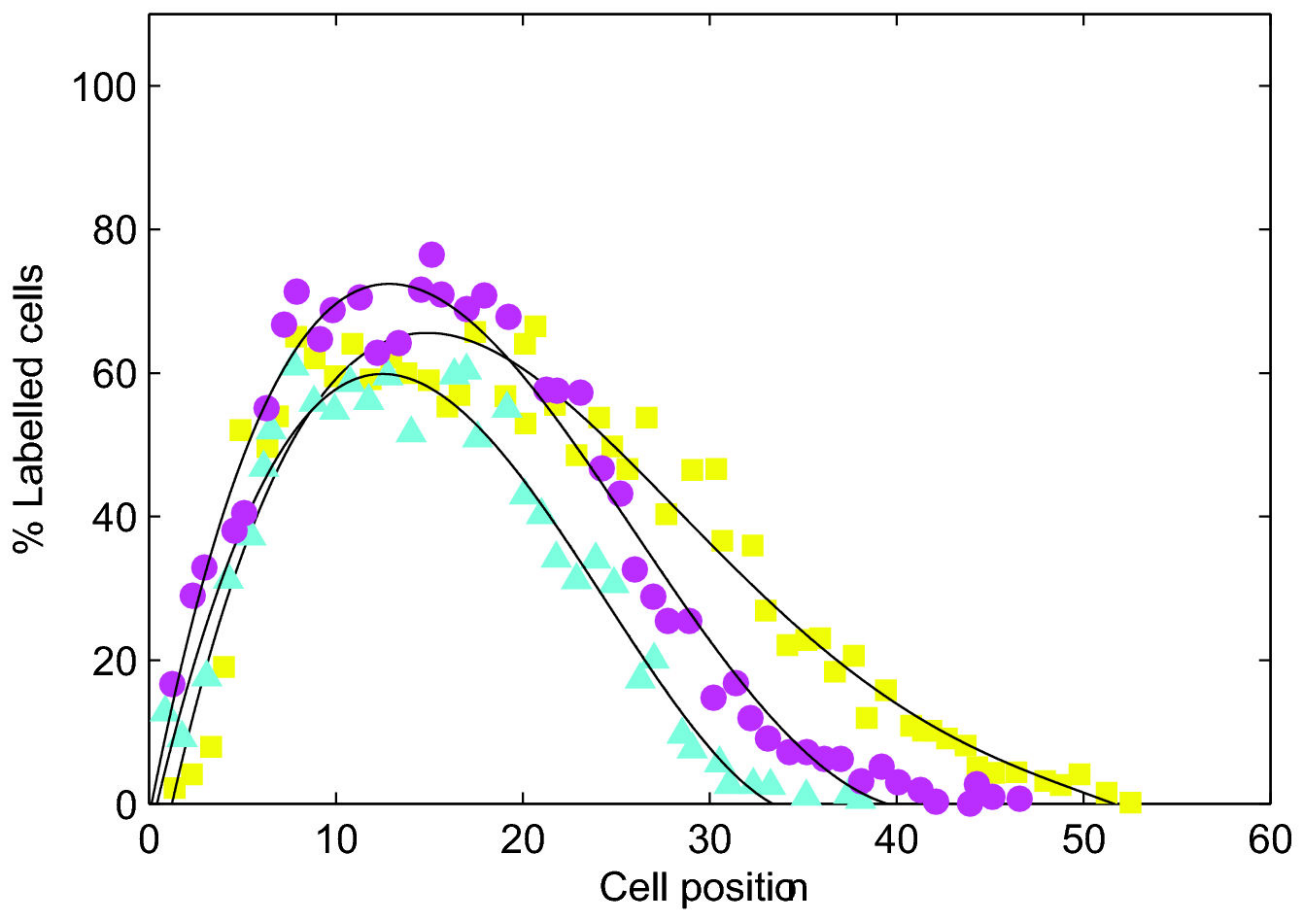
An example of experimental results showing labelled cell distributions at specific time intervals following irradiation. % Labelling index corresponds to the percentage of labelled cells in the crypt. (●) 9 hours, (▲) 12 hours, (■) 24 hours. Data re-plotted from [41].

Replicates were examined 1, 3, 6, 9, 12 and 24 hours following treatment. The change in the displacement of labelled cells was monitored using the labelling index distributions at each timepoint. Figure 6 is a re-plot of the distributions for the timepoints 9, 12 and 24 hours. In their experiments, 25 crypts were sampled per animal to produce data for 100 crypts for each group of 4 mice. Crypts were selected for scoring if they contained a sufficient number of cells (≥17 along one side) and the crypt was longitudinally sectioned [40]. Cells were numbered from the base of the crypt along one side towards the crypt collar, such that each cell position along the x-axes in Figure 6 corresponds to a single cell location. In each crypt, whether the cell in each position was labelled or not was recorded, and the results collated over all crypt samples to give the average labelling index at each cell position for the group of mice. 

The shift of the distribution curves along the longitudinal crypt axis (Figure 6) demonstrates that the labelled cells are migrating upwards over time. That continued displacement following irradiation was observed in these cells indicates that cell division is not the sole mechanism regulating epithelial cell migration in the crypt.

### Eliminating Cell Division in the Computational Models

To replicate the biological experiments *in silico*, each model was first run to a steady state and proliferative cells were labelled, then mitosis was eliminated entirely. Labelling does not affect cell dynamics but, computationally, provides a method of tracking the formerly proliferative cells both for simulation data and visualisation. Labelled cells are coloured blue in all simulation snapshots while differentiated cells are still coloured pink, and the stromal cells in the cross-sectional model are still coloured green.

Fifty simulations were run and average results considered to ensure that the results are representative of model behaviour. The behaviour of the crypts was monitored for 24 cell hours after mitosis was halted. Although migration was observed in the models over periods longer than 24 hours, this time frame was chosen to be consistent with the experimental study.

**Figure 7:**
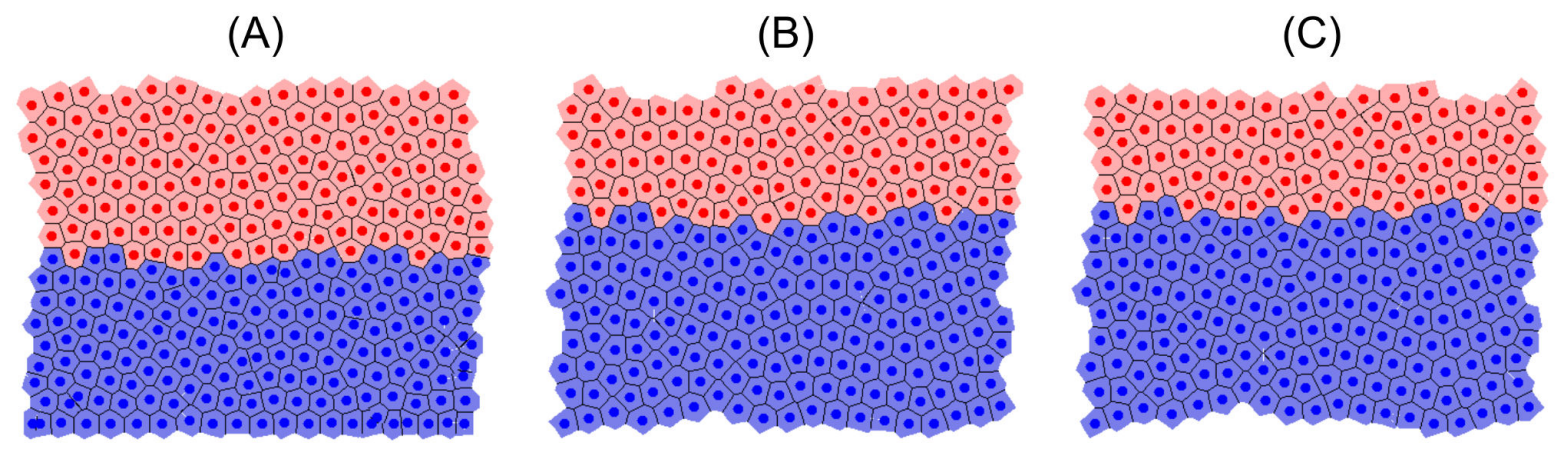
Snapshots of the cylindrical crypt model following elimination of cell division. (A) At 0 hours; the blue cells are those which were originally proliferative but are now labelled, (B) 12 hours later, (C) 24 hours later.

**Figure 8:**
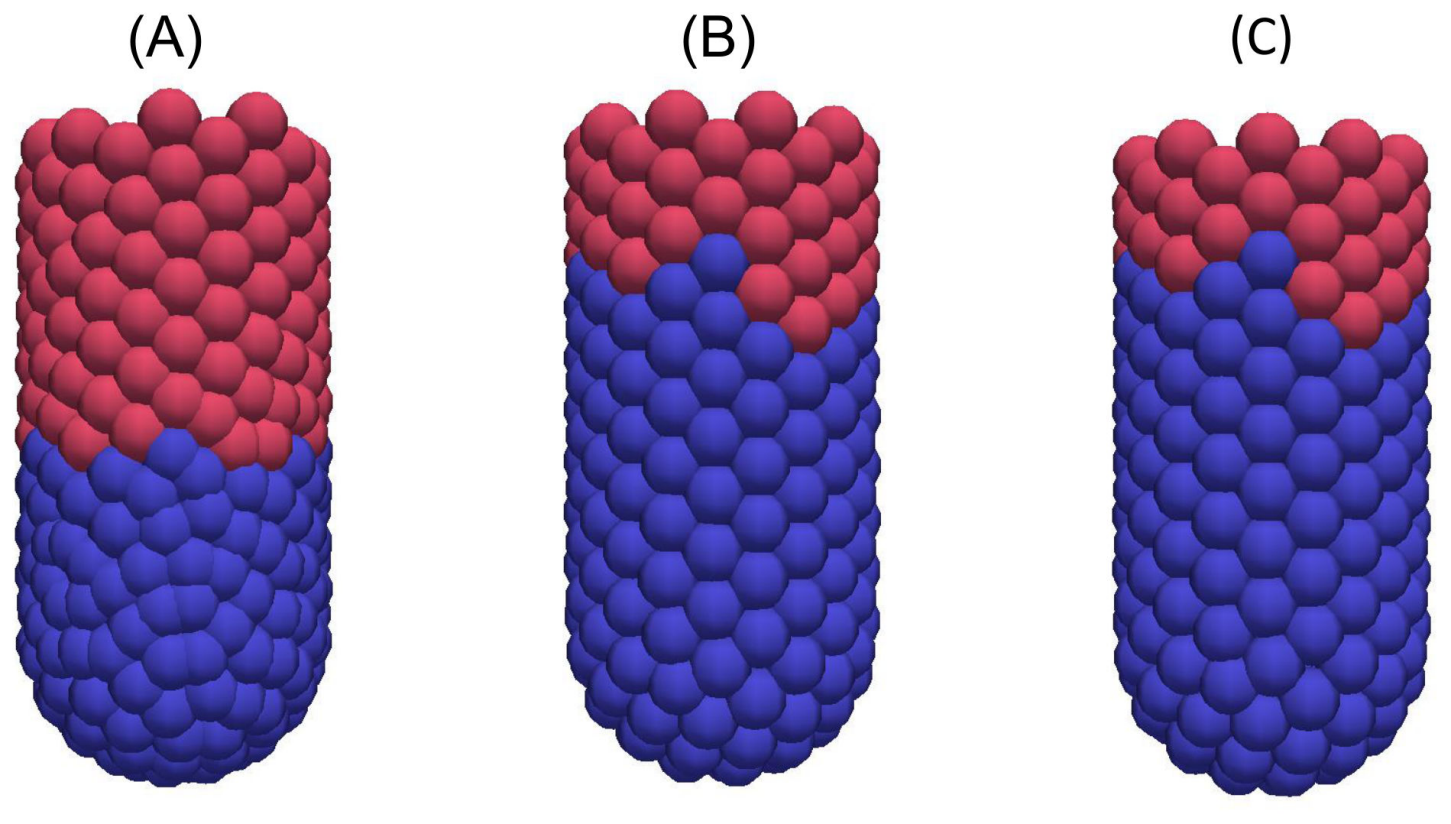
Snapshots of the 3D crypt model following elimination of cell division. (A) At 0 hours, (B) 12 hours later, (C) 24 hours later.

**Figure 9:**
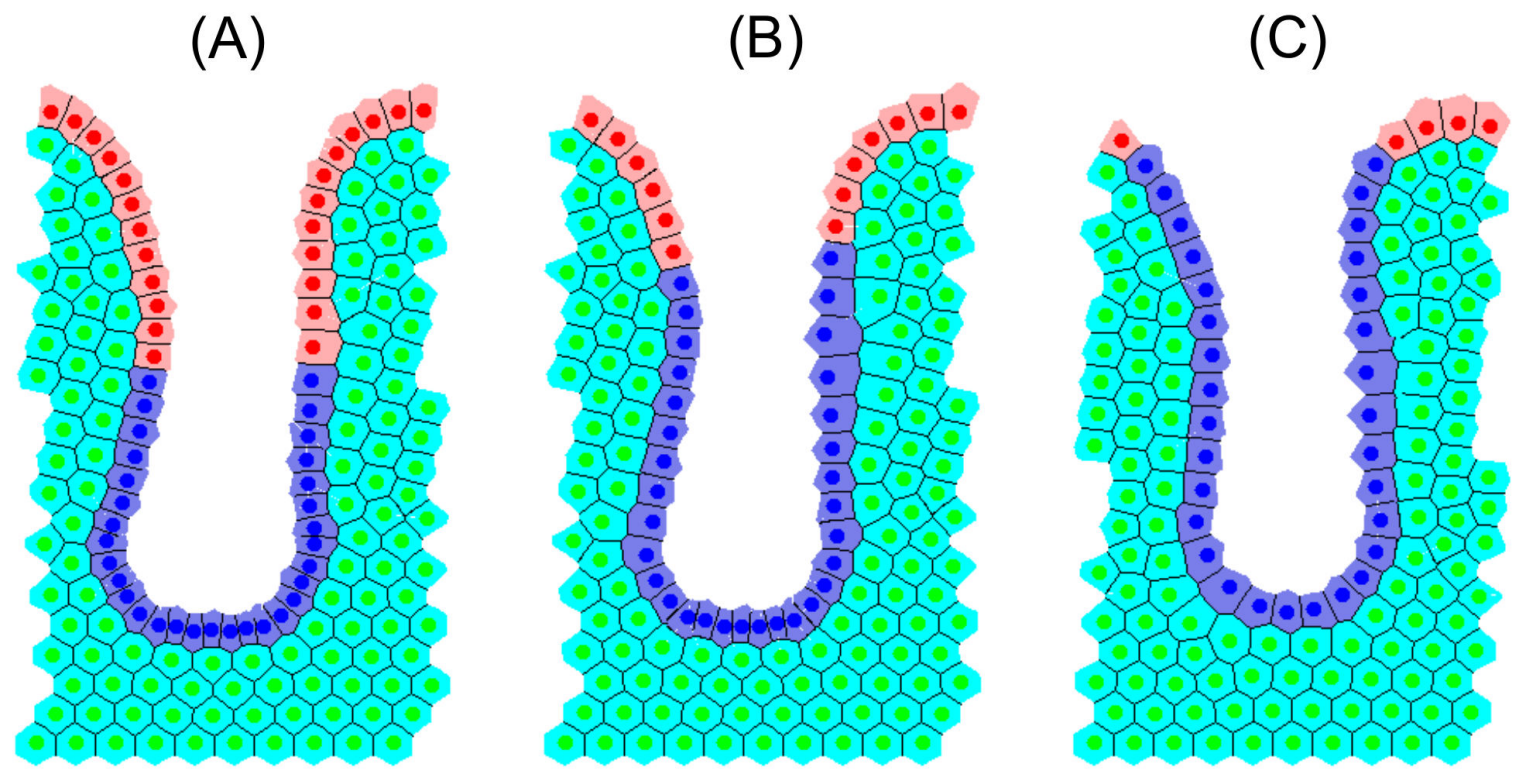
Snapshots of the 2D cross-sectional crypt model following elimination of cell division. (A) At 0 hours, (B) 12 hours later, (C) 24 hours later. These snapshots highlight that the crypt becomes shorter over time.

Figures 7, 8 and 9 are snapshots from each of the *in silico* experiments, showing the crypts, (A) at steady state with the cells that were formerly proliferative now labelled and coloured blue, (B) 12 hours later, and (C) 24 hours later. In each case, labelled cells move along the vertical crypt axis over the course of 24 hours. These images also show that the majority of cell migration occurs during the first 12 hours following treatment. Simulation movies S1-S3 of these experiments for each model are provided in the Material.

### Cell Number

**Figure 10:**
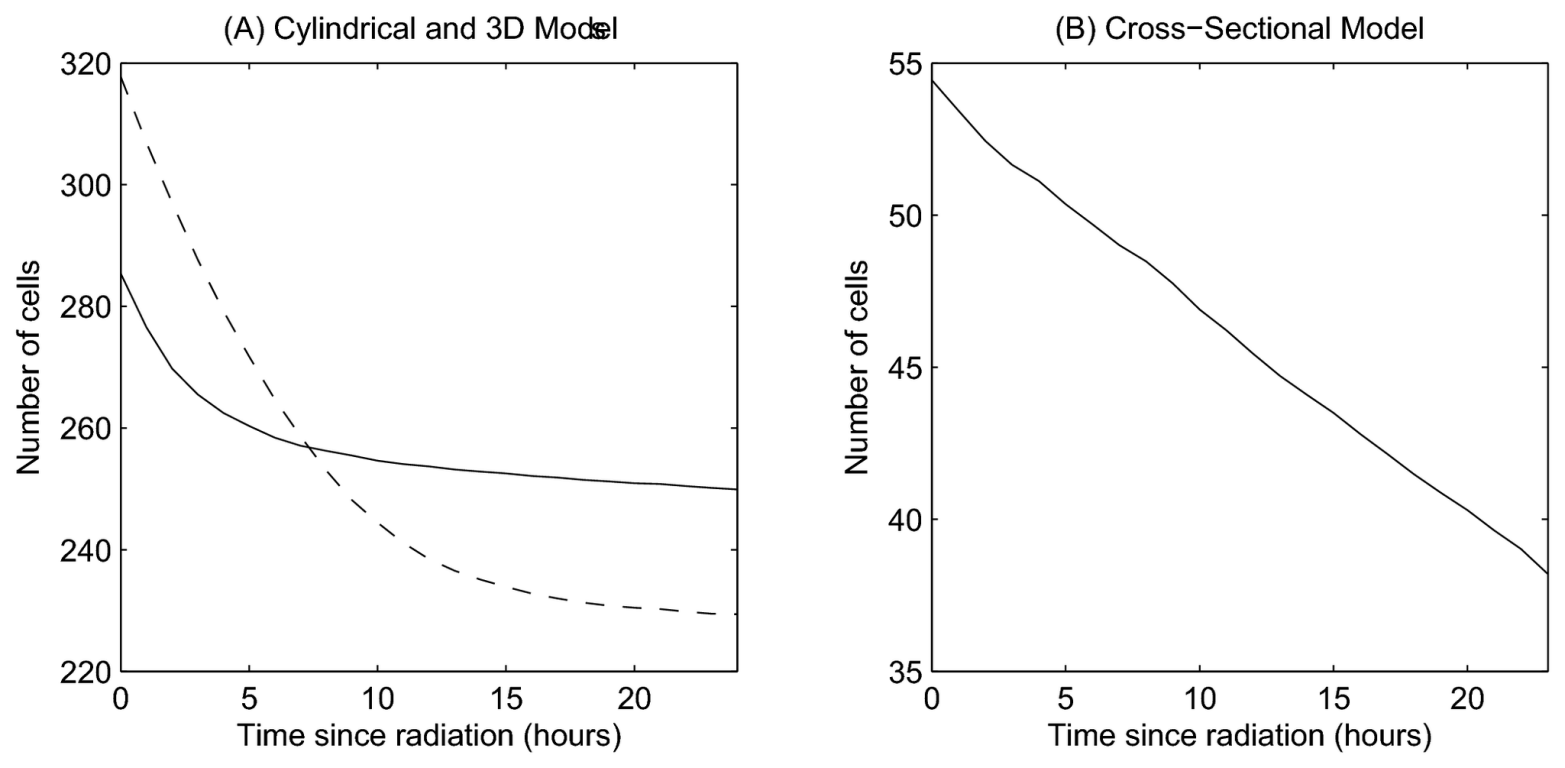
The change in epithelial cell number following elimination of cell proliferation. (A) Comparing the 2D cylindrical model (solid line) and the 3D rigid model (dashed line), (B) the cross-sectional model.

Figure 10 shows the total epithelial cell number at each timepoint following elimination of division. A change in behaviour has occurred following interference (compare with Figures 3(A) - (C)). Now, the epithelial cells steadily decrease in number due to continued cell sloughing and apoptosis events in the absence of proliferation. 

In the case of the 2D cylindrical model and 3D rigid model (Figure 10(A)), cell number decreases exponentially before plateauing, whereas a linear decrease is observed for the 2D cross-sectional model (Figure 10(B)). This difference arises due to the two types of cell death that are included in the cross-sectional model: (1) random cell death occurs in differentiated cells with a small, constant probability to capture cell death attributed to damage from passing food and waste (equivalent to sloughing in the other two models); (2) cell extrusion occurs when cells lose contact with the basement membrane at the curve of the crypt collar: anoikis [8]. The random cell death events cause cell loss to continue even when cells have fully equilibrated. In the other two models, cell sloughing at the collar requires cells to move above a threshold height, and it is not possible to model cell extrusion due to the fixed geometry. As a consequence, cell loss decreases and halts as the cells reach their equilibrium size. 

A much larger relative reduction in cell number is observed in the 3D model compared with the cylindrical model. This is due to the greater compression present at steady state (Figure 4(B)), which is subsequently relieved as cell migration and sloughing allow the crypt to relax. The decrease in cell number observed for each model following the elimination of mitosis is in agreement with experimental results which show that the crypt as a whole shrinks in size over the first 15 hours following irradiation [43]. This is evident in the deformable cross-sectional model, which is observed to shrink (Figure 9). It is not possible to observe such effects on the crypt structure in the other two models, as they each have a fixed geometry. 

### Cell Size

**Figure 11:**
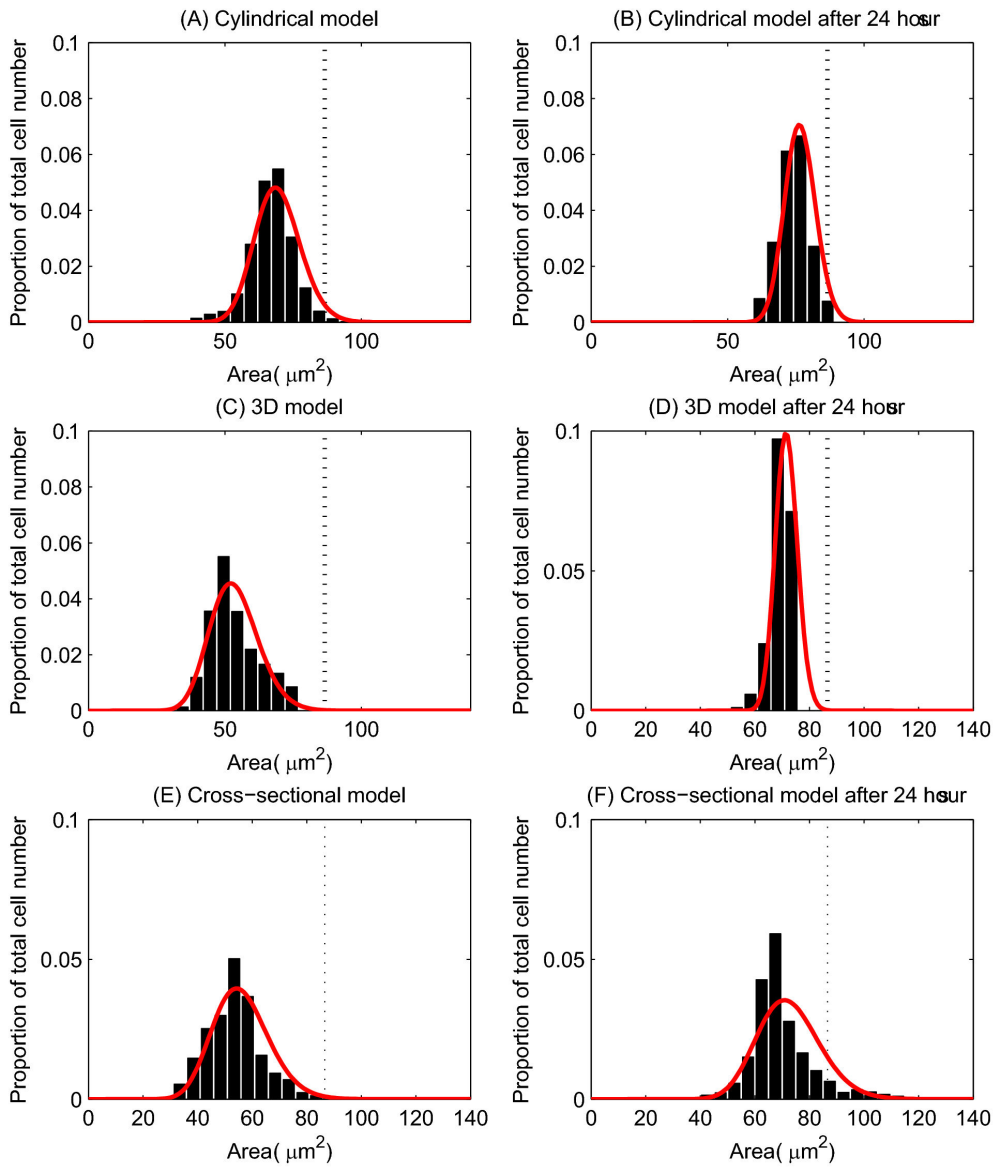
The change in the distribution of cell sizes following elimination of cell division. (A) and (B) correspond to the cylindrical model; (C) and (D) correspond to the 3D model; (E) and (F) correspond to the cross-sectional model. The left column indicates the distribution of cell areas for the model at steady state, and the right column shows the distribution of cell areas 24 hours post irradiation. The red curves mark the fitted Gamma distribution. The equilibrium cell are (86.6 μm^2^) is indicated by the vertical dotted line on each plot.

Figure 11 illustrates how the distribution of the epithelial cell areas changes after proliferation is halted. The left column in the figure corresponds to the crypt at steady state, and the right column corresponds to the crypt 24 hours after irradiation. These plots show that after 24 hours, in all models, the distributions have shifted towards the equilibrium cell sizes, indicating that the epithelial cells have relaxed and cell sizes have increased. This occurs because the compression in the layer reduces over time as the crypt relaxes and cells are sloughed from the top without cell division contributing new cells to the monolayer. In particular, this can clearly be observed in the cross-sectional model by comparing the cell sizes in Figures 9(A) and (C). It is likely that in the context of the whole tissue, these numerical changes in size translate into changes in cell shape, with cells becoming more elongated. 

### Labelled Cell Distributions

**Figure 12:**
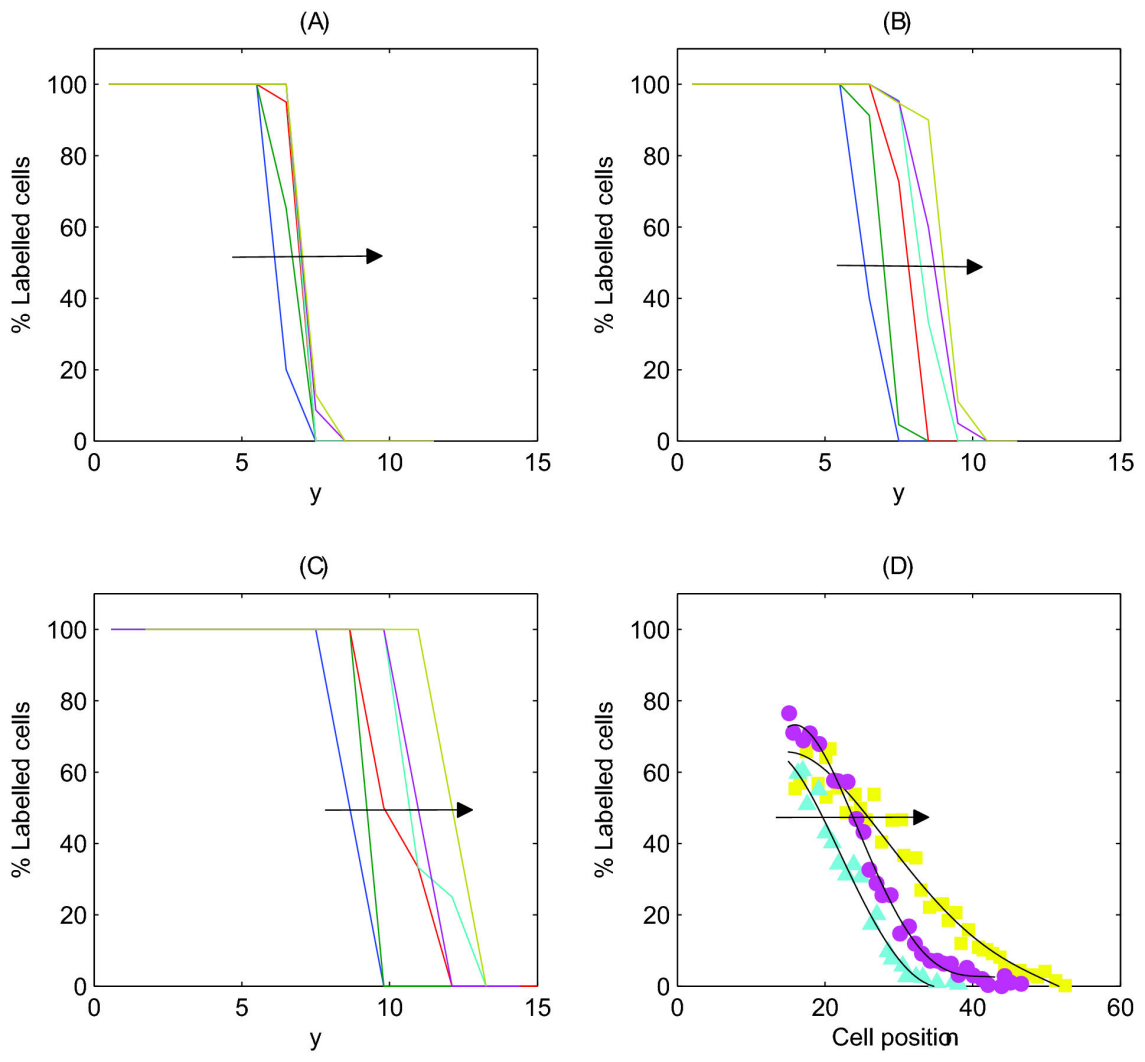
The change in the distribution of labelled cells following the elimination of cell division. Each curve corresponds to the percentage of labelled cells within horizontal bands of width 1 cell diameters after 1 hour, 3 hours, 6 hours, 9 hours, 12 hours and 24 hours (averaged over 50 simulations in each case). The arrow indicates the movement of the distribution with increasing time. Plot (D) shows the results found by Kaur and Potten for 9, 12 and 24 hours, edited from Figure 6(B) to facilitate comparison with *in* *silico* results.

To compare the results of these *in silico* experiments directly with those presented by Kaur and Potten, Figure 12 shows the labelled cell distribution, averaged over 50 simulations, at timepoints 1, 3, 6, 9, 12 and 24 hours following treatment for each model. The plots track labelled cells within horizontal sections of width equal to 1 cell diameter. Note that while Kaur and Potten use the cell position as a metric for distance from the crypt base, a measure obtained from fixed crypts, we use the distance measure *y* explicitly, due to the evolving cell sizes and positions throughout simulations. The same trend is observed in each case: labelled cells move upward along the longitudinal crypt axis over time (as indicated by the arrows), demonstrating cell migration in the absence of mitosis. 

The difference between the shape of the distributions in Figure 12 and those found by Kaur and Potten re-plotted in Figure 6 is due to the initial locations of the labelled cells. In the computational models, the labelled cells are all those that were formerly proliferative at steady state, i.e. the lower half of the crypt. Therefore, in Figure 12, 100% of cells will be labelled in the (initially) proliferative compartment, and 0% in the (initially) differentiated compartment. This leads to the observed step shape. In the experimental set-up, only a subset of the proliferative cells were labelled, specifically those in S-phase, using ^3^HTdR. This explains the different shape of the distribution. To facilitate comparison with the experimental results, Figure 12(D) shows an edited version of Figure 6, which highlights the shift of the tail of the distribution 9, 12 and 24 hours post treatment. 

In both the 3D and cross-sectional models, the distribution moves over a larger range than in the cylindrical model. In the 3D model, this is due to the greater cell compression that exists before mitosis is halted. In the cross-sectional model, because the epithelial monolayer is a 1D chain of cells, movement of an individual cell is directly influenced by the cell immediately above it in the chain. As a result, apoptosis and cell extrusion at the collar have a knock-on effect on cells below, creating a negative pressure that induces upward movement.

Overall, these *in silico* experiments agree with the results from by Kaur and Potten, and demonstrate that epithelial cells in the crypt can continue to migrate in the absence of mitosis. However, we can deduce that movement occurs due to the relaxation of initially compressed cells, as they expand into the space created by cell removal at the crypt collar. This effect relays downwards and allows cells below to expand to achieve their equilibrium size (Figure 11). Crucially, this movement occurs over the same time period investigated experimentally, and without assuming any form of active migration force.

## Discussion

Biological insight is increasingly being provided by computational and mathematical modelling. Modelling provides tools to unlock the complexity of biological data that is available, and this requires effective and active communication between biologists and computational scientists. Computational simulation adds a vital dynamic dimension to mathematical models, allowing the straightforward definition and repetition of experiments, without any dependence on animal models and the limits associated with them. Moreover, it is possible to infer mechanism from the observed behaviour of a model more readily than through experimental observation of a biological system, as the modeller knows precisely the components which constitute the model design. Of course, the extent of complexity inherent in most biological systems makes it difficult to create complete *in silico* equivalents of *in vivo* systems. Yet, a model as complex as the biological system itself would be just as difficult to interrogate, and would not expose redundancy. This underlines the need to make viable abstractions, and base computational models on as much biological data as appropriate. Only then will models generate predictions which can be investigated or invalidated *in vitro* or *in vivo*, and thus enter a feedback loop of experiment and model refinement to further understanding of the system.

The intestinal crypt is a prime example of a tissue to which mathematics and computation can contribute solidly, as many elements of its biology are well characterised, and it is a small, compact system with well-defined function. Here, three computational models were compared and used to simulate biological experiments that were performed to investigate the dependence of cell migration in the crypt on mitosis [41]: a 2D cylindrical model [10]; a 3D rigid, test-tube shaped model (inspired by [35]); and a 2D deformable cross-sectional model [34]. Each model is defined to have cell numbers based on experimental data for murine crypts, and simulations demonstrate the expected dynamic behaviour of the crypt under normal conditions: a steady turnover of cells and upward migration towards the crypt collar with increasing velocity. 

The subtle differences that arise between the models can be attributed to the geometric constraints. These results emphasise the need to choose the right model for the question that is being asked. For example, only the cross-sectional model permits investigation of cell extrusion from the epithelial layer, and accounts for the intercrypt table – where the epithelial monolayer curves towards the horizontal and the upward cell velocity decreases (Figure 5). The critical differences between the models, and how they compare to experimentally-observed behaviour, are summarised in Table 2.

Using murine crypts, Kaur and Potten [41] found that over a monitored period of 24 hours, epithelial cells still migrate along the longitudinal crypt axis despite mitotic arrest induced by radiation - it was thus concluded that cell migration cannot be solely dependent on mitotic pressure, but that a form of active migration may exist [39]. The motivation for re-visiting these experiments computationally is to investigate whether the results obtained can be attributed to the basic mechanical components of the crypt: cell division, simple adhesion and repulsion between neighbouring cells, and cell loss by apoptosis and extrusion.

*In silico* experiments show that each computational model predicts the same result: cell migration is observed following the elimination of cell division. Importantly, in all cases, an active migration force does not need to be included. After eliminating cell division, cell number decreases and cell size increases. Based on this we conclude that cells relax and expand into the space created by cell loss at the crypt collar (or villus tip), which allows the cells to move upwards. Additionally, in tissue, migration may not only occur as the cells relax, but also as a consequence of merging cell columns which would cause crypts to narrow - a similar hypothesis was proposed by Loeffler et al. [44]. It is not possible to investigate this hypothesis in a 2D model, but it would be possible in a deformable 3D crypt model; however, such a model has not yet been realised computationally. It is possible to observe that the decrease in cell number in the deformable cross-sectional model accompanies a reduction in crypt length. This has been observed experimentally following irradiation and was attributed to near-normal cell migration occurring when mitosis has stopped [43]. It is not possible to track shape changes in the crypt structure in the other two models, because cell geometries are fixed in these cases.

Cell migration eventually slows for both the 2D cylindrical and 3D models (Figures 7 and 8) and there is little change in the leading edge of labelled cells after twelve hours. This suggests that mitotic pressure is required to maintain cell movement indefinitely. Given that apoptosis also occurs at the collar in the cross-sectional model (distinct from extrusion events) the reduction in cell migration is less pronounced (Figure 9). However, it is unlikely that cells will continue to migrate indefinitely without further mitotic events - there simply would not be enough cells in the crypt to prevent loss of barrier function. It is more likely that in response to halted mitosis over times that exceed 24 hours, crypts would shrink in size.

It should also be noted that in the biological experiments, the mitotic index remained close to zero only for the first 12 hours after treatment. It is likely that the mitotic events that began to occur after this time contributed to the observations of cell movement. In the *in silico* experiments, on the other hand, mitosis was completely eliminated. Nonetheless, continued migration was still observed. Together with results from earlier investigations using the cross-sectional model [34], this suggests that cell division and apoptosis collaborate to support cell migration in the crypt, and that cell removal at the collar is a driving force. Importantly, we show that cell movement does not require additional signalling gradients, beyond that which determines cell division, or an explicit active migration force. However, crypts do contain gradients of a number of signalling molecules and are also exposed to contractile forces, which could affect migration. Thus detailed experimental work will be required to measure the contribution and influence of such factors.

The results presented here suggest that cell movement can be induced mechanically by negative pressure created by cell loss near or at the crypt collar. To investigate this experimentally, future biological experiments could utilise specific toxins to induce cell shedding/apoptosis along the crypt axis and determine the consequence on migration patterns. Inhibiting stretch-activated ion channels reduces the incidence of both apoptotic and non-apoptotic cell extrusion events that are part of normal epithelial homeostasis [8]. Therefore, inhibiting these channels is predicted to lead to a reduction in migration. In the longer term, using such treatments concurrently with inhibiting mitosis could be help to dissect the individual contributions of these processes to migration.

Further *in silico* experiments could advance to utilise a more realistic model of cell shape, as well as the development of a 3D deformable crypt model. This would permit modelling how rearrangement of cell columns and affects crypt length and shape. In addition, these models could be extended take account of differential cell adhesion mediated by Eph/ephrin signalling, to investigate the how cell sorting / positioning interact with mechanical sources of migration.

None of the computational models considered here explicitly take account of cell shape. Instead, in the 2D models, cell shape is dictated by the relative distance between neighbouring cell centres, and in the 3D model, cells are represented by overlapping spheres. *In vivo*, cells in the crypt are columnar (Figure 1). Therefore representing cell shapes as cylinders or ellipsoids would be more realistic. To increase the similarity between the models and physiological situation, adaptive cell shapes could be modelled using the subcellular element method, in which each cell is a construct of a number of subcellular elements that correspond to portions of the intracellular cytoskeleton [45]. Intra- and inter-cellular interactions are determined in conjunction with a weak stochastic component, and together these forces contribute to the motion of each element. To successfully adopt this approach, it will be necessary to make force measurements that allow us to estimate the new model parameters.

The work presented here highlights that the role of mitosis and cell death in cell migration in the crypt is yet to be fully understood, and justifies the hypothesis that simple mechanical effects are key to cell movement. The predictions provided by the three separate *in silico* models provide the basis for further investigations into the underlying mechanisms that govern crypt dynamics and this will be crucial to understand changes that commonly accompany diseases of this tissue. 

## Materials and Methods

Figure 2 shows snapshots of each computational model considered here: a 2D cylindrical model [10]; a 3D rigid, test-tube shaped model (inspired by [35]); and a 2D deformable, cross-sectional model [34]. The details of these models can be found in the original publications, but the necessary details are repeated here for the benefit of the reader. All model parameters are provided in Table 1, and the key differences between the models are summarised in Table 2. Using three different models to investigate the inhibition of mitosis demonstrates that common results are model independent. Without loss of generality, here only the crypts are considered, and not the small intestinal villi. The villi could be incorporated into these models for a future study. Unless stated otherwise, model parameters are assumed to be those stated in the original publications that detail each model.

### Model Set-Up

The dimensions of each model have been defined in accordance with murine crypt data obtained from the jejunum, about 50% into the small intestine (unpublished data [46]). Seven samples at age 60 days were used to measure 350 crypts. On average, cells were arranged into intercalated columns of approximately 15 cells, with a cross-sectional circumference of approximately 20 cells, generating approximately 300 cells per crypt. The size of the crypt domain in each model is defined according to these data, and the models can be considered as approximately equivalent in cell number and size when run to a homeostatic steady state (hereafter just referred to as a steady state) where proliferation, migration and cell death are balanced. 

Proliferative and non-proliferative epithelial cell compartments are defined according to a stochastic Wnt-dependent cell cycle model. Stem cells are not explicitly considered here, as we are interested in the effect of inhibition of mitosis, and therefore it is not necessary to delineate dividing cell types. A linearly decreasing gradient of Wnt signalling is defined along the long crypt axis (Figure 1), from 1 at the crypt base to 0 at the crypt orifice, and a threshold of Wnt is prescribed, *W*_*T*_, such that epithelial cells positioned in the top half of the crypt do not divide. For each proliferating cell, the duration of the G_1_ phase of the cell cycle is sampled from a Uniform *X*~*U*(1,3) distribution, while the remaining phases are held constant such that the total cell cycle length is between 11-13 hours [47,48]: S phase of 5 hours, G_2_ phase of 4 hours and M phase of 1 hour.

### The 2D Cylindrical Crypt Model

The 2D cylindrical crypt model (Figure 2(A)) is a lattice-free model in which cells are represented by their centres, which are points that are free to move in space [27]. Cell-cell connectivity is determined by a Delaunay triangulation of the cell centres: each triangle in the mesh is defined so that no other cell centre sits within the circumcircle of that triangle's vertices [49]. Cell shapes are prescribed by a Voronoi tessellation [50], which is the dual tessellation of the Delaunay triangulation and defines cell boundaries as the region of space around the cell centre that is closest to it rather than any other cell centre. This recapitulates the hexagonal packing typically observed in epithelial sheets. The crypt geometry is approximated as a cylindrical surface, with dimensions chosen so that there are approximately 15 cell rows and 20 cell columns at steady state, which is cut open and rolled out to form a periodic, rectangular domain. 

Interactive cell forces, which mimic cell-cell adhesion and limited compressibility between neighbouring cells, are modelled using a Hookean linear spring force, which acts along the lines defined by the Delaunay triangulation. Let $r_{i}$ be the position of cell centre *i*. The total force acting on each cell is thus calculated as the sum of the contributing forces from the springs connecting it to all neighbouring cells in the set *S*_*i*_:

$$F_{i}(t)=\mu \sum_{j\in S_{i}}{\hat{r}}_{i,j}(t)(s_{i,j}(t)-|r_{i,j}(t)|),$$

where $r_{i,j}$ is the vector from cell centre to cell centre *j*, ${\hat{r}}_{ij}(t)$is the corresponding unit vector, *s*_*ij*_(*t*) is the natural length of the spring connecting cell centres *i* and *j*, which increases from 0.3 to 1 over the first hour of the cell cycle [28], and µ is the spring constant. By neglecting inertial terms relative to dissipative terms, the velocity of cell centre *i* is given by

$$\eta_{i}\frac{dr_{i}}{dt}=F_{i}(t),$$

where *η*_*i*_ is the drag coefficient for the motion of cell centre *i*. As we consider cells to be uniform, *η*_*i*_=*η* for all *i*. By iterating in small time intervals, cell positions are updated at each timestep using the Forward Euler method. Parameters used are given in Table 1.

Periodic boundary conditions are imposed on the vertical edges to represent the unwrapped nature of the domain. Cells cannot move below the horizontal boundary of the crypt base, and those cells that move above a given threshold height,*H*_*T*_, are assumed to be sloughed from the top of the crypt, and are immediately removed from the simulation.

### The 3D Crypt Model

The 3D crypt model (Figure 2(B)) is also a lattice-free model and assumes a fixed, test-tube shaped geometry with approximately 15 cell rows and 20 cell columns at steady state. Cells are constrained to lie on this fixed geometry, while cell-cell connectivity is defined by the off-lattice over-lapping spheres model (as described in [31,32,35]), such that two cells are determined to be neighbours if the cell centres are within a maximum interaction radius of 1.5 cell diameters. Cells experience forces due to nearest neighbours, also calculated using the Hookean linear spring force implemented in the cylindrical model, along the vectors that connect neighbouring cell centres. Sloughing at the crypt collar occurs beyond a threshold height, *H*_*T*_, where cells are immediately removed from the simulation. This model is defined to be the 3D analogue of the 2D cylindrical model, and shares identical parameters with respect to cell-cell forces.

### The 2D Cross-Sectional Crypt Model

The 2D cross-sectional model (Figure 2(C)) implements a deformable basement membrane model within an off-lattice cell-centre framework in a cross-sectional crypt geometry [29,34]. While the epithelial cells are still coloured yellow and pink, the surrounding tissue stroma is approximated as a collection of cells, coloured green. 

To compare with the 2D cylindrical model and 3D rigid model, the cross-sectional model is defined such that there is a chain of approximately 50 epithelial cells at steady state (to account for the curved nature of the domain). Interactive cell forces are again defined using the Hookean linear spring force, acting along the lines of the Delaunay triangulation. Periodic boundary conditions are imposed along the vertical edges of the tissue stroma, to mimic the presence of neighbouring crypts. 

The model accounts for the supportive structure of the surrounding musculature and pericryptal fibroblast sheath by defining the basement membrane to have a non-zero spontaneous curvature at the crypt base, and a zero spontaneous curvature everywhere else. The curvature of the epithelial monolayer is used to calculate a restoring force acting on each epithelial cell centre *i* subject to each cell *j* in the neighbouring set of stromal cells, *N*_*i*_: 

$$F_{i}=\beta \sum_{j\in N_{i}}(\kappa_{ij}-\kappa_{s}){\hat{u}}_{ij}.$$

Here, β is the basement membrane force parameter, which characterises the strength of adhesion of the epithelial layer to the basement membrane and the stiffness of the membrane itself, ${\hat{u}}_{ij}$is the unit vector from the stromal cell *j* to the epithelial cell *i*, and *κ*_*ij*_ is the calculated local discrete curvature for this cell pair. The value of the spontaneous curvature, *κ*_*s*_, is dependent on where the cell pair is positioned – a non-zero spontaneous curvature is assumed at the crypt base, with a zero spontaneous curvature everywhere else. For more details of the basement membrane model, see 34.

In addition to Wnt-dependent proliferation, density-dependent inhibition of mitosis prevents division for those cells that are smaller than a defined threshold area, *A*_*T*_. Furthermore, two mechanisms of cell death are implemented. Firstly, explicit cell loss is defined to occur randomly within the differentiated cell compartment towards the crypt collar. This is defined to occur only in the top 10% of the crypt, the explicit height of which varies as the crypt grows / deforms over time, and the probability of death in one hour is 0.1. Secondly, anoikis follows cell extrusion if an epithelial cell loses contact with the basement membrane, which will occur throughout the whole crypt [8,51,52]. These mechanisms of cell loss add to the degree of stochasticity to the model, in contrast with the cylindrical and 3D models, in which stochasticity only arises due to the cell cycle.

### Simulations

All model simulations and *in silico* experiments are conducted within the Chaste framework [53], which can be accessed from http://www.cs.ox.ac.uk/chaste. The code that is used to run these simulations is released and thus available to download from the Chaste website. Chaste is an open source software library, written in object-oriented C++ and constructed using agile programming techniques. As Chaste is used to simulate each model, and the software is comprehensively tested, differences that arise in model behaviour do so because of the models themselves, and not due to differences in the simulation framework. 

### Calculating Cell Area or Volume

In the 2D models, cell area is calculated according to the Voronoi tessellation that defines the individual cell boundaries. Specifically, it is calculated as the sum of the area of the triangles contained within the polygon shape, which are formed by joining the adjacent cell vertices to the cell centre. It should be noted that the cross-sectional area to which this value refers differs between the 2D models. Given that the cylindrical model is equivalent to an unwrapped crypt, cell area corresponds to the cross-sectional cell area parallel to the basement membrane, i.e. the apical surface area. In the 2D cross-sectional model, the cell area is taken as the longitudinal cross-section, i.e. the lateral surface of the cell in contact with neighbouring epithelial cells. The data have been dimensionalised by assuming that 1 cell diameter corresponds to 10 μm [14,54]. Thus, in 2D, the equilibrium cell area is 86.6 μm^2^, which is calculated as the Voronoi area when the neighbouring cell centres are 1 cell diameter apart, and therefore exerting zero force.

In 3D, the cell volumes are approximated by taking the average of the interaction distances between each spherical epithelial cell and its overlapping neighbours, and using this distance as the effective radius of the epithelial cell of interest, *r*. The volume is then calculated as that of a sphere of radius *r*. The equilibrium cell volume is calculated as the volume of an un-deformed sphere (1 cell diameter), which is approximately 524 μm^3^. 

Given the difference in dimension between the models, we must consider cell size using the metric of cross-sectional area in 2D, and volume in 3D. For comparison, we have calculated the equivalent circular cross-sectional area for those cells in the 3D model from the volume measurements, and these results are presented in Figures 4 and 11.

## Supporting Information

Movie S1 **A movie of the 2D cylindrical crypt model, where firstly the model is run at a steady state for 24 hours (the first ten seconds of the movie), and then mitosis is halted and the simulation continues for a further 24 hours.** This allows the viewer to compare the model behaviour with and without mitosis. Proliferative epithelial cells are coloured yellow, differentiated epithelial cells are coloured pink and labelled cells (those which were proliferative before mitosis was halted) are coloured blue. Movies S1-S3 illustrate the *in* *silico* replication of the Kaur and Potten experiments: each shows the behaviour of the crypt model both at steady state, and then following elimination of mitosis, at which point the yellow proliferative cells turn to blue, and division is halted.
(MPEG)

Movie S2 **A movie of the 3D crypt model, where firstly the model is run at a steady state for 24 hours, and then mitosis is halted and the simulation continues for a further 24 hours.** Proliferative epithelial cells are coloured yellow, differentiated epithelial cells are coloured pink and labelled cells (those which were proliferative before mitosis was halted) are coloured blue.
(MPEG)

Movie S3 **A movie of the 2D cross-sectional crypt model, where firstly the model is run at a steady state for 24 hours, and then mitosis is halted and the simulation continues for a further 24 hours.** Proliferative epithelial cells are coloured yellow, differentiated epithelial cells are coloured pink, stromal cells are coloured green and labelled cells (those which were proliferative before mitosis was halted) are coloured blue. Grey cells are epithelial cells which undergo apoptosis randomly towards the crypt orifice.
(MPEG)
